# Functional Morbidity Following Latissimus Dorsi Flap Breast Reconstruction

**Published:** 2014-05-01

**Authors:** Susan L. Smith

**Affiliations:** UF Health Cancer Center at Orlando Health, Orlando, Florida

## Abstract

According to the National Institutes of Health, more than 230,000 new cases of breast cancer will be diagnosed in 2014 alone. Following mastectomy, several reconstructive options exist for women with breast cancer. The timing and approach for reconstruction must be addressed rapidly. Although abdominal tissue transfer is described as the preferred method, it may not be best suited to all patients. The latissimus dorsi (LD) muscle flap is a widely available, proven, and reliable modality. The majority of studies support that shoulder functional morbidity is minimal, but this should be more accurately quantified to allow patients to assess the possible impact on their daily lives. A critical appraisal of the available evidence was undertaken to determine the incidence of new functional morbidity involving the ipsilateral arm following LD pedicled flap breast reconstruction. The process for identifying articles included preappraised and secondary literature sources published between 2005 and 2013. Randomized controlled trials, evidence-based practice, clinical guidelines, and systematic reviews were the quality filters applied. This literature review confirmed that LD muscle transfer does lead to measurable reductions in shoulder joint stability, strength, range of motion, and general functionality. However, these deficiencies resolve in the vast majority of women within 6 to 12 months. Ultimately, the consequences of shoulder function morbidity must be considered and discussed with patients prior to making a final decision.

According to recent estimates from the National Institutes of Health (NIH), over 230,000 new cases of breast cancer will be diagnosed in 2014 alone (NIH, 2013). These patients will be forced to make immediate life-altering decisions regarding surgical intervention for treatment. Timing and approach for reconstruction must also be addressed rapidly. Eligibility is dependent upon a multitude of variables, including (a) comorbid medical conditions (de Oliveira, Pinto e Silva, Costa Gurgel, Pastori-Filho, & Sarian, 2010), (b) age, (c) weight, and (d) breast size (Kim et al., 2013). Each option has unique implications for patients and their families to consider, including disruption in employment and child care obligations, among others.

Crucial discussions take place during a rapid succession of appointments that may include seeing a general surgeon for mastectomy, a medical oncologist, a radiation oncologist, and a plastic surgeon for reconstruction. The overload of information combined with the intense emotional burden can be overwhelming. It is imperative for the patient to receive and consider comprehensive information concerning possible risks associated with various forms of breast reconstruction.

The latissimus dorsi (LD) flap is considered the reliable "workhorse" for breast reconstruction (Koh & Morrison, 2009). Its use has been described in a variety of reconstructive settings since 1906 (Hamdi et al., 2008). Other autologous tissue transfer options are associated with a prolonged period of convalescence as well as possible flap and donor site complications (Hankins & Friedman, 2008). The majority of available studies support the fact that shoulder functional morbidity is minimal, but this should be more accurately quantified to allow patients to assess the potential impact on their daily lives.

The procedure may be performed immediately following mastectomy or delayed. The LD is dissected along with a "paddle" of vascularized muscle (thoracodorsal artery and vein) overlying fat and skin (musculocutaneous flap). Once elevated, the LD is tunneled subcutaneously under the axilla and transposed into the breast pocket, where it is sutured into place. The LD restores volume to the breast pocket and is often further augmented with implants or fat grafting to provide adequate symmetry and cosmesis (Kim et al., 2013; Hankins & Friedman, 2008; de Oliveira et al., 2013).

Comprehensive patient-centered preoperative counseling and surgical planning mandates an answer to the following question: "In female breast cancer patients with acquired deformities of the breasts resulting from mastectomy, what is the incidence of new functional morbidity involving the ipsilateral arm following latissimus dorsi flap breast reconstruction?" To accomplish this, an evidence-based practice literature review was performed. Indications for LD reconstruction, appropriate patient selection, various operative techniques, timing of surgery, and presence of adjuvant therapies were considered. Timing and methods for assessment of shoulder function with documentation of complications and quantification impairment were compared.

## Methodology

The process for identifying articles included the following preappraised and secondary literature sources: The Cochrane Library, Ovid Databases (PubMed and Medline), Database of Abstracts of Reviews of Effects (DARE), Turning Research Into Practice (TRIP), NIH Public Access, Embase, Cumulative Index to Nursing and Allied Health Literature (CINAHL), and SUMSearch2. Search queries incorporated PICO (Patient, Intervention, Comparison, Outcome) terms as follows: (latissimus dorsi) AND (breast OR breast reconstruction) AND (function OR shoulder OR movement). See Table 1 for a summary of PICO criteria. For the advanced searches, query limitations were set as "human" and published between 2005 and 2013. Such a broad time range was deemed appropriate due to the paucity of relevant literature. No limitations for language or full text were selected. Randomized controlled trials, evidence-based practice, clinical guidelines, and systematic reviews were the quality filters applied.

**Table 1:**
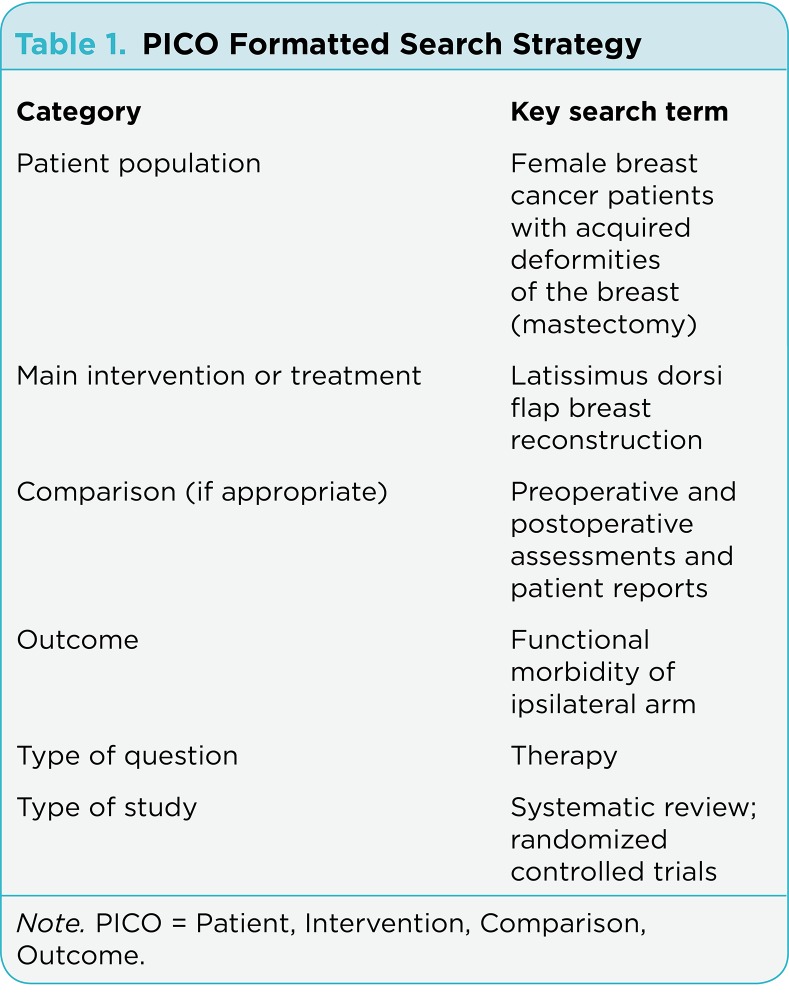
Table 1. PICO Formatted Search Strategy

Search results from SUMSearch2 yielded 34 results, the largest yield of all the databases. This included duplications from all other searches. All selected article references were hand-searched and yielded one study for inclusion. Unfortunately, the majority of articles focused on cosmetic outcomes and morbidity related to postmastectomy scars and seroma formation without describing morbidity involving shoulder function. Several only alluded to changes in shoulder function/strength without directly addressing it. Ultimately, 13 articles were selected.

Selection criteria focused on articles with study populations including women with mastectomy defects related to breast cancer treatment who underwent breast reconstruction using the LD muscle flap. Articles including patients receiving LD reconstruction for other diagnoses in both adult men and women (those over 18 years old) were included to confirm functional morbidity not occurring as sequelae to breast cancer treatment. Studies selected detailed quantitative functional and patient-reported qualitative outcomes measures. Information on study objectives, patient population characteristics, surgical interventions, assessment methodology, and study results were abstracted by the author. See Table 2 for a synopsis of the studies reviewed.

**Table 2:**
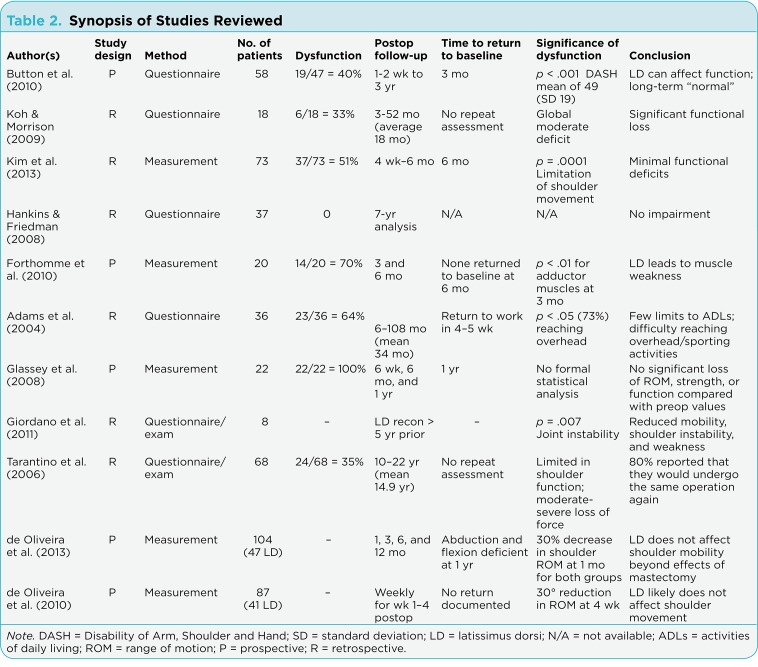
Table 2. Synopsis of Studies Reviewed

## Review of the Literature

Spear and Hess (2005) performed a literature review to identify biomechanical and functional changes in the shoulder following transfer of the LD muscle for breast reconstruction. The purpose was to search available studies to identify any clinically relevant findings for inclusion in discussions with patients considering breast reconstruction options. In spite of the methodologic differences identified, these authors found adequate evidence that some degree of weakness results from the transfer of the LD, but range of motion was not significantly affected. Based on their findings, this review suggests that patient education include expected limitations for the first 2 to 3 weeks postoperatively, with full return of function in 6 to 12 months.

Adams et al. (2004) sought to retrospectively review patient-reported outcomes following LD transfer, focusing on activities of daily living, work, and leisure. After identifying 85 consecutive female patients who had undergone this procedure, researchers mailed questionnaires seeking subjective recollection to evaluate a variety of areas. These included (a) cosmesis, (b) weakness, (c) numbness, (d) time to return to work, (e) change in vocation, and (f) duration of physical therapy. Patients were asked to rate limitations in multiple activities of daily living. A total of 36 breast reconstruction patients completed the survey. Results revealed that at least half of all patients experienced a prolonged sensation of back tightness and numbness, likely scar-related as a result of mastectomy. In addition, activities of daily living that require lifting or pushing and leisure activities, such as swimming and golf, may be more difficult. Length of follow-up did not appear to impact results.

Tarantino, Banic, and Fischer (2006) sought to identify long-term complications related to flap- and prosthetic-based breast reconstruction with a retrospective review of female patients who underwent LD reconstruction in combination with silicone implant placement, 90% of whom were diagnosed with breast cancer. Data were collected through interviews, questionnaires, clinical examinations, and photography. Of the 68 patients, 61 were diagnosed with breast cancer. The most significant and recurrent complication was capsular contracture related to the implants, which occurred in 50% of the patients. Shoulder dysfunction was reported as moderate to severe (1 in 3 patients) and interfering with daily activities (1 in 5 patients). Surprisingly, this did not decrease overall satisfaction, as approximately 80% of the women reported that they would undergo the same procedure again and recommend it to others.

Hankins and Friedman (2008) described their own experience with bilateral breast reconstruction using LD over a period of 7 years. The article began with a detailed description of the operative procedure. In this population of 37 women who underwent reconstruction using the LD flap, 27% received chest wall irradiation prior to the procedure. There were no subjective reports of diminished shoulder function or strength.

The Disability of Arm, Shoulder and Hand (DASH) questionnaire was used by Koh and Morrison (2009) to evaluate reported outcomes following LD reconstruction in a diverse group of breast, limb, and head and neck patients. This tool assesses the ability to perform activities of daily living, as well as (a) pain, (b) numbness, (c) weakness, and (d) impact on social activities. Impairments were reported pertaining to ability to perform (a) sports, (b) gardening, and (c) housework. This study included 25 participants, but only 18 completed the questionnaire. Results revealed especially high DASH scores in 33% of patients, with life-altering dysfunction experienced by some. The most intrusive morbidity appeared to be related to overhead activities.

DASH scores were also used by Button et al. (2010) to prospectively compare LD reconstruction performed using quilting and nonquilting techniques. Quilting is a suturing technique that progressively brings deep-tissue edges together, eliminating "dead space" and reducing the likelihood of seroma accumulation (Button et al., 2010). This method may lead to decreased seroma formation, but it was not associated with differences in shoulder dysfunction. Based on initial observations, the focus shifted to examining shoulder morbidity alone. The study alluded to an association between physiotherapy and positive functional outcomes. Reported findings of meaningful shoulder limitations for 6 to 12 weeks after surgery reached a plateau between 3 and 6 months, with most reporting normal function in the long term.

Kim et al. (2013) compared morbidity between two groups of breast reconstruction patients with similar demographics and sample sizes (37 and 36, respectively) who received different types of LD flaps. The extended latissimus dorsi (ELD) includes additional fat, and the muscle-sparing flap (MSLD) includes only a small strip of muscle. Limitation of shoulder range of motion was assessed with the assistance of physical therapists. Alterations were noted in both groups, yet MSLD was shown to be associated with significantly less morbidity (*(p* = .0001).

In a 2011 study from Finland, Giordano et al. evaluated shoulder function for long-term disability occurring at least 5 years after LD breast reconstruction. One goal was to eliminate the impact of breast cancer treatment by selecting a population of patients who received LD reconstruction for other soft-tissue defects, including head and neck and trauma reconstruction. The small sample of eight patients underwent physical assessments to measure range of motion and function. Patients were also asked to complete questionnaires. Subjective and objective impairment in mobility, stability, and strength were evaluated. When operated and nonoperated sides were compared, passive range of motion impairment was statistically significant for adduction and internal rotation only, and active range-of-motion was statistically significant for forward elevation as well as external and internal rotation.

A 2008 study by Hamdi et al. challenged the standard technique for performing LD harvest and proposed the use of the thoracodorsal perforator free flap—harvesting only a skin/fat paddle (fasciocutaneous tissue) and blood vessels—as an option for sparing the LD muscle itself (Hamdi et al., 2008). The patients all received breast-conserving surgery with some form of lymph node dissection and adjuvant radiation. Muscle strength and mobility were documented along with LD thickness measurements. The results indicated that the muscle-sparing free flap offers a viable alternative with significantly less functional morbidity. However, the 22 patients were not directly compared with patients receiving standard LD surgery, and forward elevation was still found to be statistically significant (*(p* = .041).

The prospective assessment by Glassey, Perks, and McCulley (2008) included 22 breast patients followed for 1 year postoperatively. The advantage of this design was that the ipsilateral side was evaluated throughout; not surprisingly, when reconstruction involved the dominant hand, these patients were slower to recover. There was an unanticipated and unexplained finding of increased range of motion to the affected side postoperatively. Although no meaningful decreases in strength and function were detected, it was found that the entire year was needed to return to presurgery functional levels.

Forthomme et al. (2010) also completed a prospective study. Twenty Belgian females who underwent unilateral mastectomy and LD flap reconstruction were evaluated preoperatively and then again at 3 and 6 months postoperatively. Their hypothesis focused on the validity and reproducibility of isokinetic muscular evaluation. Results described fatigue with overhead activities (primarily when the dominant hand side was involved) but no occupational problems. The authors concluded that muscle weakness associated with LD transfer should be anticipated and rehabilitative therapy ordered proactively.

de Oliveira et al. (2010), in the first of two studies, designed a prospective study including 87 Brazilian women comparing functional outcomes between immediately reconstructed and unreconstructed women who had undergone modified radical mastectomies. The only factors correlated with reduced abduction were (a) smoking, (b) the presence of axillary cords, and (c) axillary lymph node dissection. Latissimus dorsi reconstruction did not contribute to functional morbidity.

Noteworthy methodologic challenges were noted. The significant ethical dilemma of denying reconstruction eliminated the possibility of randomization. Another confounder was that it appeared that surgeons were preferentially selecting younger, healthier patients to undergo reconstructive procedures.

Finally, immediate breast reconstruction with LD was reexamined by de Oliveira and colleagues (2013) for recovery of shoulder range of motion during the first year following mastectomy. A primary goal was to extend the follow-up period of the preceding study. In this study, the potential effects of chemotherapy, radiotherapy, and postsurgical complications were considered. The sample size of 104 postmastectomy females was assigned according to whether or not they underwent immediate LD reconstruction. Baseline shoulder function was assessed, and all patients received physical therapy beginning on the first postoperative day. Tissue adhesion and scar formation from mastectomy alone were associated with functional limitations. There was no clinically significant functional morbidity associated with LD reconstruction.

## Discussion

Shoulder function occurs as the result of a complex interplay of 26 separate muscles. The latissimus muscle, one of the body’s largest muscles, works in concert with six additional muscles to assist the shoulder with medial rotation, abduction, shoulder extension, depressing of a raised arm and downward rotation of the scapula. Secondary function involves support of humeral head stability (Spear & Hess, 2005; Forthomme et al., 2010; Giordano et al., 2011; Glassey, Perks, & McCulley, 2008; de Oliveira et al., 2013). The remaining muscles are reportedly capable of assuming function, with only limited residual functional sequelae reported (Glassey, Perks, & McCulley, 2008; Fort- homme et al., 2010).

Approaches to the assessment of shoulder range of motion, strength, and function vary widely throughout the literature. Forthomme et al. (2010) compared isokinetic measurements preoperatively and postoperatively, between operative and nonoperative sides, for 6 months. Two related prospectively designed studies assessed patients’ range of motion 1 day prior to surgery and postoperatively for up to 1 year (de Oliveira et al., 2013; de Oliveira et al., 2010; Glassey, Perks, & McCulley, 2008). Two studies were carried out prospectively and assessed function; preoperative assessment was not performed (Kim et al., 2013; Giordano et al., 2011). A retrospective review of patients treated in the 10 to 22 years prior to these studies that revealed significant patient-reported loss of shoulder function with more significant penetrance involving over one-third of patients (Tarantino, Banic, & Fischer, 2006).

Self-report questionnaires provided qualitative assessments of strength, documenting return to work as well as ability to perform activities of daily living (Adams et al., 2004). Two studies used the DASH questionnaire to assess patient assessment of shoulder impairment following LD breast reconstruction. Results from both revealed that morbidity can be substantial but that duration of disability can differ (Koh & Morrison, 2009; Button et al., 2010). Contrary to other studies, Hankins and Friedman (2008) reported in their 7-year review that there were no subjective complaints of impaired shoulder mobility or weakness.

Physical therapy (PT) played a significant role in the care of breast cancer patients undergoing LD reconstruction. Objective measurements of range, strength, and function were performed by physiotherapists in all studies that obtained quantitative measurements. Patients received PT for differing time frames, including preoperatively and then postoperatively for up to a year, in inpatient and outpatient settings (de Oliveira et al., 2013; de Oliveira et al., 2010; Glassey, Perks, & McCulley, 2008; Hamdi et el., 2008). Two studies provided patient education for a self-exercise program rather than formal PT sessions (Kim et al., 2013; Button et al., 2010). Although it seems self-evident that functional outcomes would improve with a structured physical therapy regimen, whether formal or self-directed, this was not consistently included in the plan of care.

## Overview

Critical appraisal of available literature confirms that LD pedicled muscle transfer does lead to measurable reductions in shoulder joint stability, strength, range of motion, and general functionality. Limitations can significantly impact activities of daily living and leisure time pursuits for the first 3 months, but function generally begins to return to baseline between 6 to 12 months postoperatively. The majority of patients eventually regained "normal" function and reported a high level of satisfaction. Despite this finding, controversy remains.

Studies reviewed employed diverse methodologies and suffered from obvious design flaws. Sample sizes were small, and there was incongruous scheduling for follow-up evaluation. Moreover, a variety of techniques were employed to measure shoulder function with a range of equipment. Furthermore, there was no standardized assessment interval or method. Retrospective designs make accurately establishing change from baseline, especially by means of self-report, challenging. A significant number of patients appeared to be lost to follow-up. Handedness is an important concept that was not universally considered when operated and nonoperated sides were assessed. Additional considerations would include isolating for immediate vs. delayed and bilateral vs. unilateral surgeries. The current paucity of literature makes such focused reviews on a larger scale difficult.

Many potential confounders are inherent to breast cancer reconstruction, including (a) potential detrimental effects of healing surgical scars, (b) lymphedema, (c) pain, (d) sensorimotor issues, and (e) emotional concerns. Individual patient characteristics such as (a) medical comorbidities, (b) age, (c) adjuvant therapies, (d) smoking status, and (e) body-mass index can have significant impact on surgical outcome in any setting. It may be surprising to note that Button et al. (2010) confirmed that radiation is not thought to significantly correlate with functional results. Similarly, effects of axillary surgery resolved after 6 to 12 months, with the notable exception of lymphedema.

The literature supports the idea that over time and with exercise, other muscles will assume the function of the LD. Standardization of physical therapy protocols is imperative, as this appears to have a measurable positive impact. Conversely, evidence does not support the use of any differing LD harvest techniques designed to minimize scapula fat. Quilting may reduce seroma accumulation but does not impact shoulder function.

## Final Thoughts

Several reconstructive options exist for women following mastectomy for breast cancer. Though abdominal tissue transfer is currently described as the preferred method, it may not be best suited to all patients. The LD muscle flap is a widely available, proven, and reliable modality. There is no convincing evidence that its use should be limited, except possibly in the case of professional athletes. Ultimately, the consequences of shoulder function morbidity must be considered and discussed extensively with patients prior to making a final decision.

Advanced practitioners (AP) function as part of the multidisciplinary team assisting patients through their individual journeys. Regardless of oncology-related specialty, input is vital to informed decision-making. Using evidence-based knowledge of reconstructive options and the potential risks, the AP can offer advice and support founded on current best practices and individualized to the patient’s physical and emotional needs. 
